# DNA‐Nanocrystal Assemblies for Environmentally Responsive and Highly Efficient Energy Harvesting and Storage

**DOI:** 10.1002/advs.202206848

**Published:** 2023-03-22

**Authors:** Mallikarjuna Reddy Kesama, Sunghwan Kim

**Affiliations:** ^1^ Department of Physics and Institute of Basic Sciences and Sungkyunkwan Advanced Institute of Nanotechnology (SAINT) Sungkyunkwan University Suwon 16419 Republic of Korea; ^2^ Department of Biomedical Engineering Hanyang University Seoul 04763 Republic of Korea; ^3^ Department of Electronic Engineering Hanyang University Seoul 04763 Republic of Korea

**Keywords:** charge storage, DNA, environment modulation, nanomaterials, triboelectric nanogenerators

## Abstract

Natural polymer‐based and self‐powered bioelectronic devices are attracting attention owing to an increased interest in human health monitoring and human–machine interfaces. However, obtaining both high efficiency and multifunctionality from a single natural polymer‐based bioelectronics platform is still challenging. Here, molybdenum disulfide (MoS_2_) nanoparticle‐ and carbon quantum dot (CQDs)‐incorporated deoxyribonucleic acid (DNA) nanocomposites are reported for energy harvesting, motion sensing, and charge storing. With nanomaterial‐based electrodes, the MoS_2_‐CQD‐DNA nanocomposite exhibits a high triboelectric open‐circuit voltage of 1.6 kV (average) and an output power density of 275 mW cm^−2^, which is sufficient for turning on hundred light‐emitting diodes and for a highly sensitive motion sensing. Notably, the triboelectric performance can be tuned by external stimuli (light and thermal energy). Thermal and photon energy absorptions by the nanocomposite generate additional charges, resulting in an enhanced triboelectric performance. The MoS_2_‐CQD‐DNA nanocomposite can also be applied as a capacitor material. Based on the obtained electronic properties, such as capacitances, dielectric constants, work functions, and bandgaps, it is possible that the charges generated by the MoS_2_‐CQD‐DNA triboelectric nanogenerator can be stored in the MoS_2_‐CQD‐DNA capacitor. A new way is presented here to expand the application area of self‐powered devices in wearable and implantable electronics.

## Graphical abstract

High‐performative triboelectric (TENG) nanogenerators are demonstrated using a nanocomposite consisting of deoxyribonucleic acid, carbon quantum dots, and molybdenum disulfide nanoparticles. The TENG can generate sufficient electrical energy to turn on 100 light‐emitting diodes. Due to the light absorption by nanomaterials, TENG output powers can be enhanced by the light stimulation. In addition, the nanocomposite exhibits the charge storing capability.

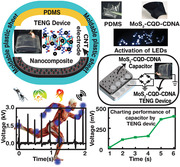

## 1 Introduction

The growing demands for green and bioelectronics have propelled the development of triboelectric nanogenerators (TENGs) that can generate a high electrical power from biomechanical motions for independent operation of bioelectronic devices.^[^ ^1^, ^2^, ^3^ ^]^ For a seamless interface with the human body, soft and biocompatible natural polymer‐TENG devices are required.^[^ ^4^, ^5^, ^6^, ^7^ ^]^ In addition, cost‐effective as well as eco‐ and biofriendly fabrication processes are important to produce reliable natural polymer‐TENG devices.^[^ ^8^, ^9^ ^]^ Therefore, natural polymers, including deoxyribonucleic acid (DNA), silk protein, cellulose, whey protein, gelatin, and egg white, are gaining considerable interest as potential materials for natural polymer‐TENGs. Additionally, efforts are being made to develop suitable fabrication processes for natural polymer‐TENGs because of their inherent biofriendly traits, which are beneficial for various applications.^[^ ^6^, ^10^, ^11^, ^12^, ^13^ ^]^ Among these natural polymers, self‐assembled DNA possess remarkable intrinsic structural, optical, electrical, magnetic, thermal, and mechanical properties.^[^ ^14^, ^15^, ^16^ ^]^ According to various reported studies, DNA has the potential to be a nontoxic and biodegradable drug delivery carrier and can be used to form optically transparent flexible films and functional layers in optoelectronic devices because of its aforementioned advantageous properties.^[^ ^17^, ^18^, ^19^ ^]^ In particular, the capability to preserve nanomaterials offers a way to expand the sphere of functionalities for DNA applications in bioelectronics.**^[^** ^20^, ^21^, ^22^, ^23^, ^24^ ^]‐^

Although the ongoing efforts on natural polymer‐TENGs are primarily focused on augmenting power generation by enhancing the triboelectrification and electrostatic induction, evaluating the TENG performance may provide a new possibility for natural polymer‐TENGs. To implement functions in natural polymer‐TENGs, incorporation of nanomaterials into natural polymer‐TENGs can be a key approach. Molybdenum disulfide (MoS_2_) is an attractive material for optoelectronic applications owing to its intrinsic direct bandgap, relative electron mobility, and decent on/off ratio.^[^ ^25^, ^26^, ^27^, ^28^, ^29^, ^30^, ^31^, ^32^ ^]^ Interestingly, in the triboelectric series, the triboelectric charging properties of MoS_2_ surpass those of polydimethylsiloxane (PDMS), making MoS_2_ a highly desirable material for TENG applications.^[^ ^4^, ^33^, ^34^ ^]^ Carbon allotropes like graphene and carbon nanotubes (CNTs) have been also applied as TENG material platforms because of their remarkable electrical properties.^[^ ^35^, ^36^, ^37^ ^]^ Among them, carbon quantum dots (CQDs) can be also used in TENGs and provide advantages of a superlight mass, smaller grains, good in solubility, ease of functionalization, photoluminescence, resistance to photobleaching (compared to its 1D counterparts), and a higher biocompatibility while maintaining a high electrical conductivity and chemical stability. The advantages facilitate numerous fascinating applications in optoelectronics, drug delivery, photovoltaics, bioimaging, and biosensing.^[^ ^38^, ^39^, ^40^, ^41^ ^]^ By incorporating the nanomaterials with DNA, multifunctional, biofriendly, and high‐performance TENGs can be developed for bioelectronic applications.

Here, we report multifunctional, high‐efficiency, and biofriendly TENGs and charge storing devices developed by assembling cetyltrimethylammonium chloride (CTMA)‐modified DNA (CDNA) structures with MoS_2_ nanoparticles (MoS_2_ NPs) and CQDs. Spectroscopic measurements (optical absorption, Fourier transform infrared (FTIR) response, and ultraviolet photoelectron spectroscopy (UPS), and X‐ray photoelectron spectroscopy (XPS)) revealed that each nanomaterial is strongly correlated in the nanocomposite. Among the tribomaterials including silk protein, skin, DNA, indium tin oxide (ITO) on glass, plastics, and metals, PDMS showed the highest output power with the MoS_2_‐CQD‐CDNA nanocomposite film. Using a repetitive contact–separation process, we obtained an average triboelectric output open‐circuit voltage (*V*_OC_) of ≈1.6 kV, an average short circuit current (*I*_SC_) of ≈3.2 µA, and a power density (*P*_out_) of ≈275 mW cm^−2^ with a load of 1 MΩ, which was sufficient for turning on 100 light‐emitting diodes (LEDs) using biomechanical motions. The performance of the MoS_2_‐CQD‐CDNA nanocomposite natural polymer‐TENG could be tuned by external stimuli, such as light illumination and thermal energy. The UV absorption by CQDs and visible to near infrared absorption by MoS_2_ NPs enable the broadband photodetection and the light‐induced enhancement of the TENG operation in the MoS_2_‐CQD‐CDNA nanocomposite film. Furthermore, the prepared nanocomposites can be used as electrical biocapacitors and connected to the natural polymer‐TENG device. Additionally, the electrical properties of the nanocomposites, such as dielectric constants, work functions, and bandgaps, were investigated. We observed that the electrical charges generated by the natural polymer‐TENG could be successfully stored in the connected biocapacitor. This fabricated TENG device can be used in small and portable self‐powered electronics. The MoS_2_‐CQD‐CDNA nanocomposite offers a versatile material platform for bioelectronics, where various electrical components such as energy sources and photodetectors can be integrated on a single platform.

## 2 Results and Discussions

**Figure 1:**
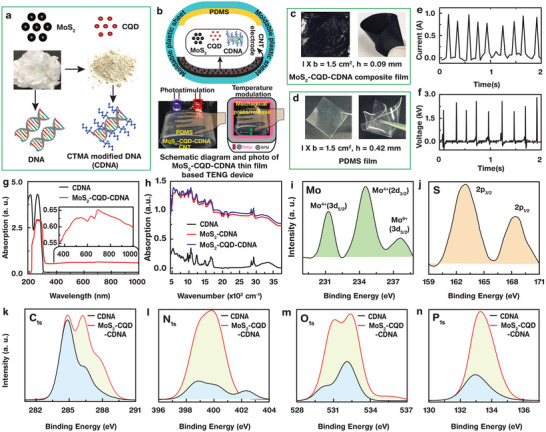
Fabrication of the MoS_2_‐CQD‐CDNA TENG device, response, and optical characterizations. a) Represented images of the MoS_2_ NPs, CQDs, DNA, and CDNA molecules. b) Schematic of the MoS_2_‐CQD‐CDNA TENG device, photostimulation, and temperature modalities setups. c,d) MoS_2_‐CQD‐CDNA and PDMS thin film size and flexibility. e,f) *I*_SC_ and *V*_OC_ of the MoS_2_‐CQD‐CDNA TENG device. g) Absorbance spectra of the pristine CDNA and MoS_2_‐CQD‐CDNA thin films. h) FTIR spectra of the pristine CDNA, MoS_2_‐CDNA, and MoS_2_‐CQD‐CDNA thin films. i–n) High‐resolution XPS spectra of the core elements (i.e., Mo, S, C, N, O, and P) present in the MoS_2_‐CQD‐CDNA thin films.

MoS_2_ NPs, CQDs, and CDNAs were used to fabricate the environmentally modulated and flexible energy harvesting/storage layer (**Figure** **1**a). To extract the generated electrical signal (electrode), a thin CNT layer was grown on the MoS_2_‐CQD‐CDNA nanocomposite layer. Subsequently, the nanocomposite layer was mounted on a moldable plastic sheet to form the PDMS layer such that both the layers faced each other (Figure 1b). These two films came in contact when a mechanical pressure was applied on them and separated upon releasing the pressure, resulting in triboelectrification. Notably, PDMS was selected because of its optical transparency and ability to generate a high TENG output power. The obtained TENG devices can be simulated by external signals such as light and heat (Figure 1b). As shown in Figure 1c,d, the nanocomposite and PDMS layers were cropped to an area of 1.5 cm^2^ and were highly flexible because of their small thicknesses. To fabricate a 90 µm thick TENG device, 0.5 wt% of MoS_2_ and CQDs were used and the resulting TENG exhibited a high and consistent electrical output power (*P*_out_), photoresponse, mechanical stability. Initially, the responses of the TENG device were examined at different MoS_2_ NP concentrations (0.1, 0.25, and 0.5 wt%) and MoS_2_‐CQD‐CDNA nanocomposite film thicknesses (10, 50, 90, 120, and 150 µm). Evidently, the TENG formed by embedding 0.5 wt% of MoS_2_ NPs and CQDs in a 90 µm thick CDNA thin film showed better and consistent results compared to the other combinations (Figures S1 and S2, Supporting Information). The nanocomposite films with thicknesses < 90 µm were very fragile under an applied mechanical pressure. Although the >90 µm thick nanocomposites exhibited a better mechanical stability, the performance of the corresponding TENG deteriorated.

For the MoS_2_‐CQD‐CDNA nanocomposite films, CNT electrodes were found to be more beneficial than the widely used silver‐nanowire‐based flexible electrodes, because the conductivity of a silver‐nanowire‐based electrode degrades as a result of oxidation and corrosion in biological environments, which limit their biological applications. This shortcoming of silver‐nanowire electrodes can be overcome via molecular passivation or by using gold nanowires. However, these processes increase the fabrication costs manifold. In contrast, CNTs can be obtained cost‐effectively and possess exceptional properties as electrode materials, including a high electrical/thermal conductivity, biocompatibility, photo/electrothermal conversion capability, and a superior mechanical/chemical stability.^[^ ^42^, ^43^, ^44^ ^]^ Finally, a high‐performance TENG device was developed, which showed a high open‐circuit voltage (*V*_OC_) and short‐circuit current (*I*_SC_) under ambient conditions, even with a relatively small contact area (Figure 1e,f).

UV–visible–near‐infrared (UV–vis–NIR) absorption spectroscopy was used to examine the binding modes and interactions between MoS_2_, CQDs, and the CDNA molecules (Figure 1g). The pristine CDNA film exhibits characteristic absorption peaks at central wavelengths of 210 and 260 nm, which originate from the ribose, phosphate, and amide groups in CDNA.^[^ ^42^ ^]^ The 260 nm peak broaden (band‐edge shift) up to ≈320 nm because of presence of conjugated C=C structures in the CQDs. The peaks at 525 (broad peak center) and 635 nm are characteristic of the MoS_2_ NPs (Figure 1g, inset).^[^ ^45^ ^]^ The observed redshift (i.e., bathochromism) and decrease (i.e., hypochromic effect) in the intensity of the absorption peak in the UV region can be ascribed to the electrostatic interactions of MoS_2_ and CQD with CDNA. Next, FTIR spectroscopic analyses were conducted to examine the molecular interactions between the nanomaterials (Figure 1h). The measured spectral profiles can be categorized into four sections: peaks corresponding to the vibration modes of the sugar and phosphate backbone groups of CDNA (500–1250 cm^−1^), peaks attributed to the vibration and stretching modes of nucleobases, MoS_2_, and CQD (1300–1800 cm^−1^), peaks ascribed to the CH**_2_** and CH**_3_** groups in the CTMA surfactant (2850−3000 cm^−1^), and peaks associated with the stretching modes of the —OH, C—H, and N—H groups (3000−3600 cm^−1^) (Table S1, Supporting Information). The vibrational and stretching mode absorption band positions and their corresponding band assignments are shown in Table S1 in the Supporting Information.^[^ ^42^, ^44^, ^45^, ^46^ ^]^ The decreased peak intensities and peak shifts confirm physical manipulation of the strong interactions among the MoS_2_ NPs, CQDs, and CDNA (compared to pristine CDNA). The MoS_2_ NPs and CQDs tend to be wrapped by the CDNA molecules, resulting in electrostatic and noncovalent interactions among CDNA, the MoS_2_ NPs, and CQDs.

High‐resolution XPS was performed to examine the functional groups, chemical compositions (and quantify them) and chemical states (associated with the binding energies) present in the MoS_2_‐CQD‐CDNA thin films. The survey spectra in Figure S3a,b in the Supporting Information show that the pristine CDNA consists of bases, sugar, and phosphate group elements (C, N, O, and P) and the MoS_2_‐CQD‐CDNA thin films consist of Mo, S, C, N, O, and P. The MoS_2_ NPs introduce Mo and S, whereas the CQDs introduce C in the nanocomposite (Figure 1i–n). The Mo spectrum exhibits three peaks at binding energies of 231.27, 234.53, and 237.63 eV, corresponding to the chemical states Mo^4+^ (3d_5/2_), Mo^4+^ (3d_3/2_), and Mo^6+^ (3d_5/2_), whereas the S spectrum shows two characteristic peaks at 163.00 and 168.40 eV, representing the 2P_3/2_ and 2P_1/2_ states, respectively.^[^ ^47^, ^48^ ^]^ The binding energy peaks (Figure 1k–n) and the corresponding functional groups (Figure S1c–j, Supporting Information) of the pristine DNA and nanocomposite are discussed in Section S2 in the Supporting Information.

When the MoS_2_ NPs and CQDs were incorporated into CDNA with chemical groups of C—C/C=C/C—H, C—O/C—N/N—C=N/N—C—N, and N—C—O/N—C=N/N—C=O, the corresponding XPS spectrum showed shifts and disappearance of the binding energy peaks (assigned to N—C(=O)—N), because the CQDs altered the chemical state of C_1s_ (Figure 1k). In the case of N_1s_, the observed binding energy peak at 402.35 eV arises from the N—C functional group (Figure 1i). In the O_1s_ state, an extra binding energy peak appears at 535.41 eV due to the C—C—P/C—C—C/P—O functional group (Figure 1m). Additionally, the binding energy peaks of all the chemical states of N_1s_, O_1s_, and P_2p_ exhibit significant intensity enhancements and shifts compared to those of the pristine CDNA (Figure 1k–n). All the observed spectral footprint changes in the XPS spectra indicate chemical interactions among the MoS_2_ NPs, CQDs, and CDNA, implying that the MoS_2_‐CQD‐CDNA nanocomposite is not a simple mixture of these three nanomaterials. Table S2 in the Supporting Information shows the appreciable changes in peak heights, full widths at half maxima, peak areas, atomic percentages, and binding energy changes of CDNA and MoS_2_‐CQD‐CDNA.^[^ ^42^, ^49^, ^50^ ^]^

**Figure 2:**
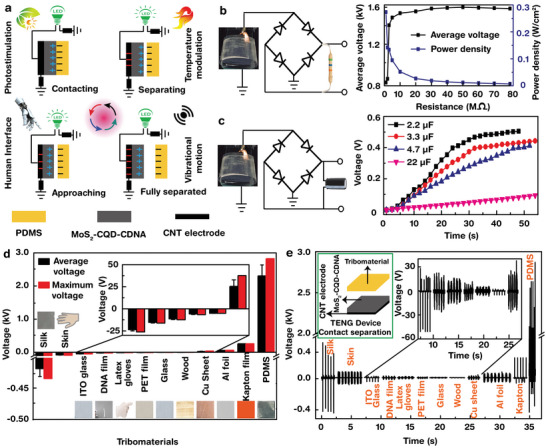
Triboelectric generation mechanism of the MoS_2_‐CQD‐CDNA TENG device and its performance. a) Schematic illustration of the triboelectric mechanism of the MoS_2_‐CQD‐CDNA TENG device involved in energy harvesting under biomechanical motions, vibrational motions, photostimulation, and temperature modalities. b) *V*_OC_ values measured with varied external load resistances and the calculated power densities. c) Charging response of commercially available capacitors using the TENG device. d,e) Average and maximum *V_OC_* generated by various tribomaterials, such as silk, skin, an ITO coated glass, a DNA film, a latex glove, a PET film, glass, wood, a Cu sheet, an Al foil, a Kapton film, and a PDMS flexible thin film and their corresponding signals.

The MoS_2_‐CQD‐CDNA nanocomposite was then used to develop a highly efficient TENG device. In the single‐electrode configuration, the nanocomposite could generate an electrical output due to triboelectric and electrostatic inductions through repetitive contact/noncontact cycles with the PDMS surface (**Figure** **2**a). The charge transfer mechanism can be explained as follows: i) Initially, when two layers come into contact, electrostatic induction occurs via transfer of electrons from PDMS (high electron affinity) to MoS_2_‐CQD‐CDNA (less electron affinity); equal and opposite charges are induced on both the surfaces, and thus, no electrical potential difference is generated in the contact condition (i.e., *V*_OC_ = 0). ii) Once the two layers are separated, the potential difference gradually increases, leading to the induction of negative charges on the buried CNT electrode; thus, electrons flow to the MoS_2_‐CQD‐CDNA nanocomposite in this case. iii) The transient electron flow continues until the two facing layers are totally separated. iv) When the PDMS layer again comes in contact with the nanocomposite layer, the process is reversed, i.e., electron flow in the opposite direction is observed. Upon repeating this process, alternative electrical outputs can be obtained. By using MoS_2_ NPs and CQDs, the output power can be enhanced via optical stimulation, which can increase the number of electron–hole pairs generated in this process.

Figure 2b shows the average *V*_OC_ values and power densities obtained from the MoS_2_‐CQD‐CDNA TENG device at various load resistances (*R*_load_). As *R*_load_ increases up to 50 MΩ, *V*_oc_ first increases and then saturates. The TENG shows the maximum output power density (*P*_out_ = (*V*_OC_^2^/*R*_load)_/*A*, where *A* was the contact area) of 275 mW cm^−2^ at *R*_load_ = 1 MΩ. The obtained *P*_out_ is exceptional when compared to those obtained from the previously reported MoS_2_‐nanocomposite‐based TENGs (Table S3, Supporting Information). The generated charges are stored into capacitors with capacitances of 2.2, 3.3, 4.7, and 22 µF (Figure 2c). The lower the capacitance, the faster the charge storage. For 30 s, the 2.2, 3.3, 4.7, and 22 µF capacitors could apply *V*s = 0.45, 1.38, 0.28, and 0.05 V, respectively. Additionally, we investigated the effect of other triboelectric (TE) materials on the electrical output (Figure 2d,e). We tested a silk protein film, human skin, an ITO‐coated glass, a DNA film, a latex glove, a polyethylene terephthalate (PET) film, glass, wood, a Cu sheet, an Al foil, a Kapton film, and PDMS. A larger electron affinity difference between two TE materials in contact result in a higher *V*_OC_ and information on this electron affinity difference indicates the compatibility of a material with our MoS_2_‐CQD‐CDNA nanocomposite platform. Evidently, the silk protein film, human skin, ITO‐coated glass, DNA film, a latex glove, PET, glass, and wood showed electron affinities less (tribopositive) than those of the MoS_2_‐CQD‐CDNA nanocomposite, whereas the Cu sheet, Al foil, Kapton, and PDMS showed more affinities (tribonegative). Due to the high contact electrification (high *V*_OC_ output), we adopted PDMS for the counterpart TE layer.^[^ ^10^, ^51^ ^]^

**Figure 3:**
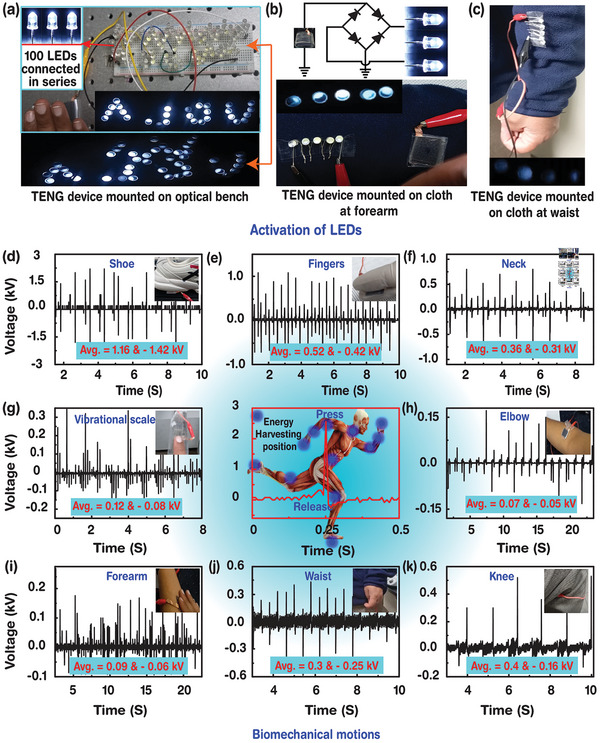
Activation of the LEDs and the MoS_2_‐CQD‐CDNA TENG device response with various biomechanical motions. a) Activation of 100 LEDs connected in series on a breadboard with a rectification bridge circuit. b) Activation of LEDs mounted on a forearm fabric. c) Activation of LEDs without rectification, where the device and LEDs are mounted on a fabric at the waist and on the forearm, respectively. d–k) Generation of output voltage signals by various biomechanical motions such as shoe, fingers, neck, vibrating scale, elbow, forearm, waist, and knee movements, respectively. The biomechanical energy harvesting device mounting positions on the human body and pressing and signal release of the MoS_2_‐CQD‐CDNA TENG device.

The electrical energy harvested by the MoS_2_‐CQD‐CDNA TENG was used to turn on LEDs (**Figure** **3**a). We made two signs using 28 and 100 LEDs connected in series, all of which could be activated by tapping on the mounted TENG (Movies S1 and S2, Supporting Information). Figure 3b shows the TENG and five LEDs that were mounted on a fabric. These five LEDs could be turned on by applying only a small mechanical force. The TENG was mounted on the fabric at the waist area, while the LEDs were mounted on the fabric at the forearm, and the ground electrode was held by bare hands (Figure 3c). The LEDs were activated by the biomechanical movement of the hand, which did not touch (side to side) any rectifier circuit, proving that different biomechanical‐touching methods can be applied to harvest electrical energy from the fabricated device. To investigate the feasibility of energy harvesting by body movements in daily life, the MoS_2_‐CQD‐CDNA TENGs were mounted on various body parts and electrical output signals were measured (Figure 3d–k). For all the points, we could obtain sensitive and high *V*_OC_ electrical responses. For instance, the TENG mounted under a shoe exhibited a *V*_OC_ of 1.2 kV (average) and 2.2 kV (maximum) for normal walking (Figure 3d). These values may increase for jumping or running, wherein more pressure is applied. Further, finger tapping, chin's up‐and‐down movements, scale vibrations, and elbow's folding could generate high *V*_OC_ values of the order of a few kiloelectronvolts (Figure 3e–h). Additionally, the TENGs were also mounted on the forearm, wrist, and knee and showed *V*_OC_ for finger‐touching and sitting (Figure 3i–k). The obtained average and maximum *V*_OC_ values are displayed as a bar graph in Figure S4 in the Supporting Information. These results indicate that the TENG has a high potential for motion‐sensing applications.

**Figure 4:**
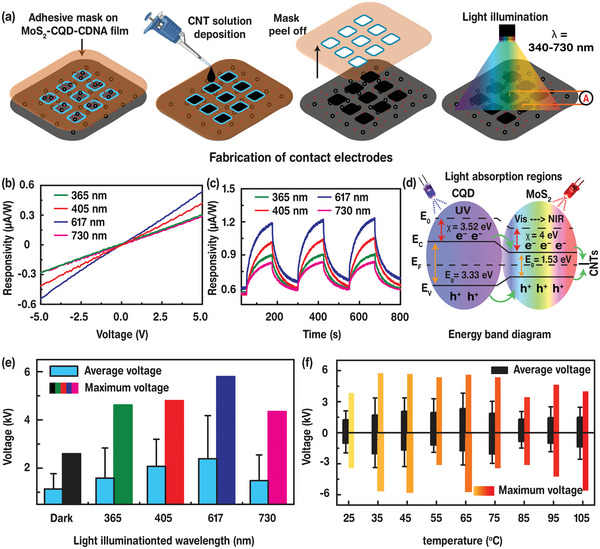
MoS_2_‐CQD‐CDNA device fabrication, analysis of photoresponsivity, and temperature effects. a) Schematic illustration of the fabrication of electrical contacts and light illumination setup of the MoS_2_‐CQD‐CDNA device. b,c) Photoresponsivity calculated at different wavelengths of 365, 405, 617, and 730 nm at a bias voltage of 5 V. d) Energy band diagram and possible charge transport mechanism under light illumination. e) Average and maximum *V*_OC_ response in dark and variation with illuminated light wavelength. f) Average and maximum *V*_OC_ response at different temperatures from 25 to 105 °C.

The electrical bandgap energies and gap energies of MoS_2_ NPs and CQDs are compatible to those visible light photons, indicating that these materials are responsive to light and generate additional charge carriers upon light illumination. The CNT electrode array was deposited on the MoS_2_‐CQD‐CDNA nanocomposite film using an adhesive tape with a 1 mm^2^ square array pattern as a stencil mask (**Figure** **4**a). Briefly, the CNT solution was dropped on the mask‐attached nanocomposite film and flattened by a flexible plastic film. Then, the sample was dried at 40 °C and the mask tape was gently detached. Two CNT electrodes were connected by wires and a multimeter to measure the electrical responses to light emitted by 365, 405, 617, and 730 nm LEDs. Figure 4b shows the current–voltage (*I*–*V*) characteristics of the device measured under both dark and light (LED illumination) conditions. Ohmic behavior (monotonical increase in the current) is visible in the range of −5 to 5 V. Although the pristine CDNA shows a conductance under the bias voltage due to charge carriers hopping through the nucleobases of CDNA (i.e., A–T and G–C intrastrand *π*−*π* coupling), the conductivity can be enhanced by the addition of MoS_2_ NPs and CQDs. Furthermore, the light exposure induces a photocurrent (*I*_p_) in the nanocomposite and results in a further enhancement in the conductivity. Electron–hole pairs were generated from the light‐absorbed MoS_2_ NPs and CQDs and transferred to the electrode by the applied electric field. Additionally, the illumination‐wavelength‐dependent *I*_p_ reveals that the nanocomposite shows a high conversion efficiency under visible light.

The time‐dependent *I*_p_ responses for repetitive light illumination are shown in Figure 4c. The device was illuminated thrice with a switching interval of 125 s and the generated *I*_p_ was recorded in real time. As the LED light wavelength was tuned, the *I*_p_ increased instantly under a 5 V applied bias, and then decayed to its initial state, when the LED was turned off. The photodetection results demonstrate a good repeatability and reproducibility. To evaluate the performance of the broadband photodetector, response time constants for the rise (*τ*_r_) and decay (*τ*_d_) of the signal were obtained (Table S4, Supporting Information). The parameters *τ*_r_ and *τ*_d_ are defined from the time taken for *I*_p_ to increase to 90% of the saturation current from 10% and that taken for *I*_p_ to decrease to 10% from 90%, respectively. The measured *τ*_r_ and *τ*_d_ values were comparable with the photodetectors integrated on the flexible substrate film.^[^ ^25^ ^]^ The low response time might be attributed to the dielectric nature of DNA and an improper band alignment further increases the scattering, thereby decreasing the mobility of the charge carriers. Figure 4d showed the energy band diagram, the charge transfer mechanism is discussed in Section S3 in the Supporting Information.

Interestingly, the photoinduced current shown by our MoS_2_‐CQD‐CDNA nanocomposite can be related to the enhancement of the TENG performance. For all the illuminated samples, the average and maximum *V*_OC_ values increase (Figure 4e). Detailed *V*_OC_ responses with respect to time are shown in Figure S5 in the Supporting Information, and the average and maximum *V*_OC_ values are indicated in Table S5 in the Supporting Information. The enhancement in *V*_OC_ originates from the additional charges generated by light. The MoS_2_ NPs and CQDs absorb the light and generate electron–hole pairs owing to the localized electric field induced by the electron excitation. Additionally, these NPs can transport the generated charges, thereby reducing the recombination loss (Figures S5–S7, Supporting Information). Higher outputs for visible light indicate the higher contribution of the MoS_2_ NPs to the charge generation.^[^ ^50^ ^]^ Our results prove, for the first time, that the TENG output can be enhanced by an external stimulus and offer new opportunities for multifunctional energy harvesting and self‐powered tactile sensing platforms suitable for wearable and implantable bioelectronics.^[^ ^52^ ^]^

The TENG performance is also influenced by temperature. We measured the *V*_OC_ at different temperatures from 25 to 105 °C with an interval of 10 °C (Figure 4f). Up to 65 °C, the *V*_OC_ gradually increases, but the trend is not monotonous. Over 65 °C, the performance drops due to thermal fluctuations; the TENG can still function, although with a lower efficiency. The real‐time TENG responses at different temperatures are displayed in Figures S8 and S9 and Table S6 in the Supporting Information. Mostly, the charge transfer or device efficiency depends on the electronegativity of the materials and area of the contacts, whereas the surface roughness, thermal fluctuations, and mechanical properties (as stiffness and ductility) of the materials are closely related to the environmental temperature. Generally, material turns stiffer and less ductile at lower temperatures, but softer and more ductile at higher temperatures. Initially at low temperatures, negligible thermal fluctuations occur, which hardly affect the separation and mobility of the charge carriers and the structural stability of the CDNA molecules. When the temperature increases, thermal fluctuations increase, leading to a weaker charge separation and eventually a smaller output. The temperature also affects the mechanical properties of the materials and friction between the layers, which are related to ductility, and the extent of microfriction between the rough surfaces of the two materials on the microscale, which is related to stiffness.^[^ ^53^ ^]^

**Figure 5:**
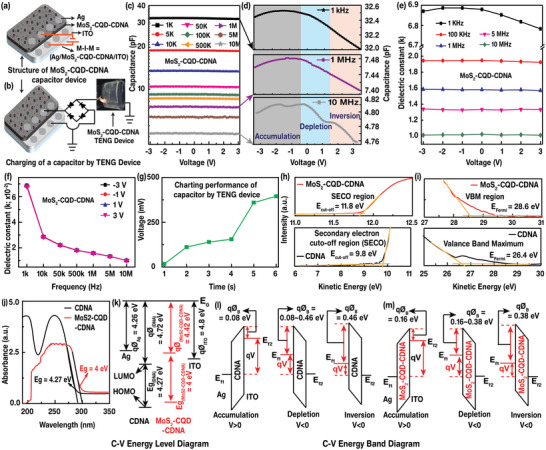
Capacitor performance. a,b) Schematic illustration of the MIM capacitor structure and its charging by the MoS_2_‐CQD‐CDNA TENG device. c) *C−V* characteristics of the MoS_2_‐CQD‐CDNA capacitor at frequencies (*f*) of 1, 5, 10, and 50 kHz, and at 0.1, 0.5, 1, 5, and 10 MHz. d) Accumulation, depletion, and inversion regions. e) Calculated dielectric constant (*k*) of the MoS_2_‐CQD‐CDNA capacitor at *f* = 1 kHz to 10 MHz. f) *k* = *k*(*f*) at a fixed voltage (−3, −1, 1, and 3 V). g) Charging of the MoS_2_‐CQD‐CDNA capacitor by tapping on the MoS_2_‐CQD‐CDNA TENG device. h,i) UPS analysis of the pristine CDNA and MoS_2_‐CQD‐CDNA thin films. j) Bandgap estimation of the pristine and MoS_2_‐CQD‐CDNA thin films. k) Energy level diagram indicates the work function (*qϕ*) of each material along with the bandgap. *E*_0_ represents the vacuum energy level. l,m) Energy band diagram of the charge transfer mechanism in the accumulation, depletion, and inversions regions. Here, *E*_f1_ and *E*_f2_ represent the Fermi energy levels, *ϕ*_B_ represents the work‐function barrier height, and *qV* represents the potential energy barrier height.

In addition, capacitance–voltage (*C*–*V*) measurements of the MoS_2_‐CQD‐CDNA nanocomposite film were conducted to investigate the interfacial trap density (volumetric energy density) and to estimate the dielectric constant (*k*) at different frequencies (*f*s) and applied voltages (*V*s), which are important to evaluate the performance of high‐energy storage devices, e.g., biopolymer‐based capacitors and batteries. **Figure** **5**a shows the schematic of the metal–insulator–metal (MIM) capacitor structure utilized by the nanocomposite; the MoS_2_‐CQD‐CDNA film was used as the insulating layer. To construct the device, a uniformly distributed MoS_2_‐CQD‐CDNA solution was coated on commercially available ITO‐glass substrates and electrical contacts were fabricated by applying silver paste on the MoS_2_‐CQD‐CDNA film; another electrode was formed on the ITO substrate.

We performed the *C*–*V* measurements at *f*s = 1, 5, 10, and 50 kHz, and 0.1, 0.5, 1, 5, and 10 MHz to investigate the *f*‐dependent capacitance. As expected, *C* monotonically decreases as *f* increases as shown in Figure 5c. Magnified plots clearly reveal the changes in the majority carriers at the interfacial layers between the nanocomposite, top (Ag), and bottom ITO conductive layers, represented by three regions: accumulation, depletion, and inversion (Figure 5d). When a high negative voltage (−3 V) is applied to the electrode, the negative charge carriers (i.e., electrons) are pushed toward ITO, and the remaining positive charges (holes) are attracted by the opposite electrode, followed by the formation of an accumulation region, where the maximum capacitance is observed. For a high positive voltage (3 V), the negative charge carriers are attracted toward the MoS_2_‐CQD‐CDNA and ITO electrode interface, which forms an inversion region. Here, more positive shifts of *C* are observed due to the depletion of holes in the region when a positive voltage is applied. At a high *f*, the capacitance behavior is clearly distinguished from that observed at low *f* values. The MoS_2_‐CQD‐CDNA nanocomposite exhibits a relatively larger *C* in the low *f* region, and it decreases at higher *f* values, because the polarity vanishes in the high *f* region.^[^ ^54^ ^]^

Additionally, the dielectric constant (*k*) of the nanocomposite was calculated as a function of the applied voltages (from −3 to 3 V) at a constant *f* of 1 and 100 kHz, and at 1, 5, and 10 MHz (Figure 5e), using the formula: *k* = *Cd*/*ε*_0_ *A*, where *d*, *A*, and *ε*_0_ are the thickness (1.5 µm), area (15 mm^2^), and vacuum permittivity, respectively. The *k* values of MoS_2_‐CQD‐CDNA decrease as the applied *f* increases, consistent with the fundamental behavior of a material (as *f* increases, the dipoles polarity vanishes). The nonlinear behavior depends on the applied bias voltage polarity (+V or −V). Figure 5f shows the *k* = *k*(*f*) values of the MoS_2_‐CQD‐CDNA film at fixed voltages of −3, −1, 1, and 3 V. The *k* value decreases for a fixed voltage as *f* is increased to 10 MHz because of the reduced interfacial charge polarization.^[^ ^55^, ^56^ ^]^

Interestingly, the fabricated Ag/MoS_2_‐CQD‐CDNA/Ag‐ITO capacitor (instead of the commercially available capacitor as shown in Figure 2c) was connected to the MoS_2_‐CQD‐CDNA TENG device with a rectification bridge circuit to verify the charging behavior (Figure 5b). As evident from Figure 5g, the Ag/MoS_2_‐CQD‐CDNA/Ag‐ITO capacitor charges rapidly by continuously tapping on the MoS_2_‐CQD‐CDNA TENG device. These results could be another evidence of real‐time energy harvesting usage of the Ag/MoS_2_‐CQD‐CDNA/Ag‐ITO capacitor and MoS_2_‐CQD‐CDNA TENG.^[^ ^57^, ^58^ ^]^

UPS analysis was performed to determine the work functions (*ɸ*s) of the pristine CDNA (*ϕ*_CDNA_) and nanocomposite (*ϕ*_MoS2‐CQD‐CDNA_) from the secondary electron cutoff region (*E*_cutoff_) and Fermi energy region (*E*_Fermi_), as shown in Figure 5h,i. The *E*_Fermi_ and *E*_cutoff_ values of MoS_2_‐CQD‐CDNA increase because of the MoS_2_ NPs and CQDs, resulting in an enhanced conductivity. Using the formula, *ɸ* = *hν* − (*E*_Fermi_ − *E*_cutoff_), where *hν* denotes the incident UV photon energy (21.22 eV), we obtained *ϕ*_CDNA_ = 4.72 eV and *ϕ*_MoS2‐CQD‐CDNA_ = 4.42 eV. The reduced *ɸ* indicates that an enhanced charge transport in the MoS_2_‐CQD‐CDNA nanocomposite, consistent with the decrease in the bandgap energy (*E*_g_) to 4 eV (MoS_2_‐CQD‐CDNA) from 4.27 eV (CDNA) deduced from the absorption spectra (Figure 5j). The unknown *ϕ*_CDNA_ and *ϕ*_MoS2‐CQD‐CDNA_ values were estimated from the UPS spectra and found to be 4.72 and 4.42 eV, respectively; the *ϕ* values of Ag and ITO are *ϕ*_Ag_ = 4.26 eV and *ϕ*_ITO_ = 4.8 eV (Figure 5k). We introduce the work‐function barrier height (*ϕ*_B_), which depends on the movement of the charge carriers in the accumulation, depletion, and inversion regions (Figure 5l,m). The flow of the charge carriers in the device strongly depends on the polarity of the applied voltage. In the accumulation condition (*V* >> 0, e.g., at 3 V), the negative charge carriers are attracted toward and accumulate at the interfacial layer between the Ag electrode and the dielectric material (MoS_2_‐CQD‐CDNA). Consequently, the potential‐energy barrier height (*qV*) decreases, followed by a reduction in *ϕ*_B_. In contrast, in the inversion condition (*V* >> 0, e.g., at −3 V) the negative charge carriers are pushed toward the ITO electrode of MoS_2_‐CQD‐CDNA, and the remaining positive charge carriers (holes) are attracted toward the negative electrode (Ag), followed by the formation of an inversion region; consequently, the *qV* (*ϕ*_B_) decreases (increases). In the depletion region (e.g., low negative voltage), the *qV* is between the accumulation and inversion values.^[^ ^23^, ^42^ ^]^

## 3 Conclusion

We report a natural polymer‐based MoS_2_‐CQD‐CDNA nanocomposite capable of high‐performance and environmentally stimulated energy harvesting and charge storage. Various spectroscopic methods, such as UV–vis absorption spectroscopy, FTIR spectroscopy, UPS, and XPS, were used to evaluate the interactions among MoS_2_, CQD, and CDNA and to estimate the bandgaps, chemical bondings, molecular structures, work functions, and charge transfer behaviors. The nanocomposite‐based TENG exhibited a high *V*_OC_ and output power, which were sufficient to turn on hundred LEDs, under biomechanical motions. Additionally, we confirmed that various biomechanical motions in daily life can induce TENG responses. Interestingly, the TENG performance could be enhanced by external stimuli such as light and heat because of the additional charges generated by the incorporated nanomaterials. The capacitances and dielectric constants of CDNA and the MoS_2_‐CQD‐CDNA nanocomposite were evaluated at different applied bias voltages and frequencies, and the obtained results revealed the interface trapping density based on the accumulation, depletion, and inversion mechanisms. Finally, the MoS_2_‐CQD‐CDNA capacitor was successfully charged by the connected MoS_2_‐CQD‐CDNA TENG. Our results open a new avenue for the development of natural polymer‐based multifunctional bioelectronic devices for soft robotics and wearable electronics.

## 4 Experimental Section

### Materials and Devices

Enzyme‐isolation processed water‐soluble salmon DNA fibers were procured from GEnome Medical (GEM) corporation, Shiga, Japan. These DNA fibers were further modified with CTMA surfactant to synthesize an organically soluble substance. The detailed synthesis of the CDNA molecules is described in Section S1.1 in the Supporting Information. The MoS_2_ NPs (size: 90 nm) and CQDs with a quantum efficiency > = 5% were purchased from Sigma‐Aldrich. The PDMS prepolymer (SYLGARD 184) was mixed with a curing agent (SYLGARD 184) purchased from Sigma‐Aldrich. Single‐wall CNTs with an average diameter of 0.84 nm were purchased from Sigma‐Aldrich. A 0.6 mm thick Cu sheet was procured from Nilaco Corporation, Japan.

### Fabrication of the MoS_2_‐ and CQD‐Embedded Self‐Supporting Flexible CDNA Thin Film

For the preparation of the MoS_2_–CQD‐embedded CDNA thin film, 0.5 wt% of MoS_2_ NPs and 250 µL of CQDs were dissolved in butanol and magnetically stirred at 1000 rpm for 5 h at room temperature (RT). The solution was then sonicated for 5 min to ensure that a homogeneous particle distribution. Next, 2 wt% of CDNA was added to the above solution and then magnetically stirred for ≈12 h at RT. Finally, the homogeneously distributed MoS_2_ NP‐ and CQD‐embedded solution was ready for preparing the flexible films. To construct the MoS_2_–CQD‐embedded self‐supporting flexible thin film (MoS_2_‐CQD‐CDNA), different volumes, i.e., 1.5, 3, 5, 7, and 9 mL (to vary the film thickness to 10, 50, 90, 120 and 150 µm, respectively) of the MoS_2_‐CQD‐CDNA mixture solution were into a 1 in. petri dish, followed by drying at 45 °C in an oven for about 3 d. The dried MoS_2_‐CQD‐CDNA thin films were gently peeled off the petri dish.

### Fabrication of the MoS_2_‐CQD‐CDNA TENG Device

The peeled off MoS_2_‐CQD‐CDNA self‐supporting flexible thin film was cut into a film of size 1.5 × 1.5 cm^2^ and a CNT solution (Section S1.2, Supporting Information) was applied to the backside of MoS_2_‐CQD‐CDNA thin film to obtain a stable conductivity. Next, the MoS_2_‐CQD‐CDNA sample was left to dry for about 1 h. A 0.6 mm thick Cu sheet was cut into small wire‐like shapes and attached on the CNT‐coated bottom electrode to capture the electrical charges generated by triboelectrification. A PDMS film (Section S1.3 in the Supporting Information) was prepared, which was cut into the same size as that of the MoS_2_‐CQD‐CDNA film (i.e., 1.5 × 1.5 cm^2^) and used as the top layer of the TENG device. Finally, the CNT bottom contacted MoS_2_‐CQD‐CDNA thin film and PDMS films were mounted opposite to each other on a moldable plastic sheet. These two films came in contact when a mechanical pressure was applied, and automatically separated when the applied pressure was released. With this configuration, the MoS_2_‐CQD‐CDNA based TENG device was ready to be used for energy harvesting through triboelectrification. For the photodetector characterization, a 1 mm^2^ CNT‐electrode‐embedded MoS_2_‐CQD‐CDNA thin film was prepared with a distance of 1 mm between the contacts. For the capacitor characterization, the MoS_2_‐CQD‐CDNA solution was drop‐cast on the ITO‐coated glass substrate, dried, and finally Ag paste was applied as the top and bottom contacts as shown in Figure 5a. This device was used to analyze the capacitor characteristics, such as *C–V* characteristics, at different frequencies and determine the dielectric constant with respect to voltage and frequency. Finally, the charging behavior of the MoS_2_‐CQD‐CDNA‐based MIM structural capacitor with time was studied by tapping on the MoS2‐CQD‐CDNA TENG device.

### Characterizations

Optical absorption studies were conducted using a Cary eclipse fluorescence spectrophotometer (Agilent technologies, Cary 5000). FTIR spectroscopy (Thermoelectric Corp., Nicolet is50) was performed to characterize the functional groups. The XPS analysis was performed using an FC‐XP10 system (Thermo Fisher Scientific) equipped with a fixed excitation source of monochromatic Al K*α* X‐rays with an energy of 1486.6 eV; this system was operated in the constant analyzer energy mode with a beam spot of ≈650 µm and an electron penetration depth of ≈10 nm. The binding energies were measured from 0 to 1350 eV to evaluate the binding energies of the pristine CDNA and MoS_2_‐CQD‐CDNA flexible thin films. UPS was performed using an FC‐XP10PS system (Thermo Fisher Scientific), equipped with a nonmonochromatic helium discharge lamp (He I) as the excitation source with an energy of 21.22 eV, to estimate the work functions of the pristine CDNA and MoS_2_‐CQD‐CDNA flexible thin films. The *V*_OC_ values of the MoS_2_‐CQD‐CDNA TENG device were recorded using a digital storage oscilloscope (Tektronix, TDS2012B), and the *I*_SC_ values were measured by a probe station (Keithley Instruments Inc., 4200‐SCS semiconductor parameter analyzer) under atmospheric pressure and RT. The transient current was measured by a current preamplifier (ITHACO, 1211) at applied bias voltages of 5 V. To evaluate the photoconductivity, the transient currents were measured under different LEDs emitting in the UV to vis–NIR regions with wavelengths of 365, 405, 617, and 730 nm. A hot plate (IKA, IKA C‐MAG HS7) was used to increase the temperature of the MoS_2_‐CQD‐CDNA TENG device, and the thermal responses were captured by an FLIR ETS320 thermal imaging camera. The *C–V* measurements were performed using a probe station semiconductor parameter analyzer (Keithley instruments Inc., 4200‐SCS).

### Statistical Analysis of the MoS_2_‐CQD‐CDNA TENG Device Performance

The *V*_oc_ and *I*_sc_ values of preprocessing data were recorded in excel form using digital storage oscilloscope (Tektronix TDS2012B), semiconductor parameter analyzer (4200‐SCS), and current preamplifier (ITHACO, 1211) instruments. The originlab software was used to plat all the graphs and Figures S6 and S9 in the Supporting Information denoted the average output voltage value lines were calculated through microsoft excel. Finally, all displayed figures were allegiantly drawn by adobe illustrator.

## Conflict of Interest

The authors declare no conflict of interest.

## Supporting information

Supporting information

Supplemental Movie 1

Supplemental Movie 2

## Data Availability

The data that support the findings of this study are available from the corresponding author upon reasonable request.
