# Supplementary material for: DNA‐Nanocrystal Assemblies for Environmentally Responsive and Highly Efficient Energy Harvesting and Storage
Source: Adv Sci (Weinh). 2023 Mar 22;10(14):2206848. doi: 10.1002/advs.202206848 (PMC10190503; doi:10.1002/advs.202206848)
Supplement: Supplementary file 1 — Supporting information [file ADVS-10-2206848-s003.pdf]

ADVANCED SCIENCE
Open Access

Supporting Information

for *Adv. Sci.*, DOI 10.1002/advs.202206848

DNA-Nanocrystal Assemblies for Environmentally Responsive and Highly Efficient Energy Harvesting and Storage

*Mallikarjuna Reddy Kesama and Sunghwan Kim**

Supporting Information

DNA-Nanocrystal Assemblies for Environmentally Responsive and Highly Efficient Energy Harvesting and Storage

Mallikarjuna Reddy Kesama and Sunghwan Kim[*]

S1. Experimental Section

S1.1. DNA and synthesis of CTMA-modified DNA (CDNA) molecules

Natural salmon DNA duplexes were extracted from a salmon fish by an enzyme isolation procedure, and subsequently dissolved in deionized (DI) water. To disperse the salmon DNA molecules in organic solvents, the DNA molecules were modified with the cetyltrimethylammonium (CTMA) cationic surfactant through an ion-exchange reaction method. In our case, 3 g of the salmon DNA molecules were dissolved in 500 mL of DI water with magnetic stirring (1000 rpm) at room temperature for 10 h. Next, 3 mL of the CTMA solution was added in another beaker, which contained 500 mL of DI water, and stirred (1000 rpm) at room temperature for ~2 h. Later, the CTMA surfactant solution was slowly added to the fully dissolved salmon DNA solution using a syringe, followed by three hours of stirring at 800 rpm. The CTMA-surfactant-modified salmon DNA molecules precipitated along with unbonded residuals such as CTMA and NaCl biproducts. The residual CTMA and NaCl biproduct were removed by a vigorous filtration process. The filtered CTMA-DNA (CDNA) paste was dried overnight at around 50 °C to obtain the CDNA powder. The CDNA powder thus prepared was ready for an organic solvent.

S1.2. Fabrication of the MoS_2- and CQD-embedded CDNA flexible thin films.

First, 0.1, 0.25, and 0.5 wt.% of MoS_2 and 0.5 wt.% of CQDs were added to a butanol solution followed by stirring (~700 rpm for 8h) to obtain a homogeneously distributed MoS_2 and CQD solution. Next, 2 wt.% of CDNA was added in each MoS_2 and CQD solution, followed by stirring (~1000 rpm for 24 h) to obtain a uniform distributed MoS_2- and CQD-embedded CDNA (MoS_2-CQD-CDNA) solution. We used the solvent evaporation technique to construct the flexible, self-supporting MoS_2-CQD-CDNA thin films. In this process, 1.5, 3, 5, 7, and 9 mL of the MoS_2-CQD-CDNA solution were drop-cast into 35-mm petri dishes, followed by drying in an oven at 45 °C for two days; the temperature was then reduced to 35 °C for one day. The dried self-supporting MoS_2-CQD-CDNA thin films were gently peeled off the petri dish. The MoS_2-CQD-CDNA thin-film thicknesses were 10, 50, 90, 120 and 150 µm, corresponding to the 1.5, 3-, 5-, 7-, and 9-mL drop-cast MoS_2-CQD-CDNA solutions, respectively.

S1.3. Fabrication of CNT ink

In this case, 1 mg. mL^{-1} of single-wall carbon nanotubes (CNTs) were well-dispersed initially in water with ultrasonication for ~2 h, and then 5 mg. mL^{-1} of sodium lauryl sulfate surfactant and 3 mg. mL^{-1} of polyethylene glycol were added to prevent aggregation and obtain a viscous solution, respectively. This final solution was continuously stirred to obtain a homogeneous distribution of the CNTs.

S1.4. Preparation of the PDMS thin film

For preparing the polydimethylsiloxane (PDMS) flexible thin film, the prepolymer and curing agent were well mixed in the ratio of 10:1 ratio, and then the solution was placed in a refrigerator to remove air bubbles. Subsequently, the mixture was poured onto a petri dish and spin-coated at 500 rpm for 2 min. The resulting PDMS film was heated on a hot plate at 60 °C for ~4 h for

curing. Finally, the solidified PDMS thin film was peeled off the petri dish, and its thickness was measured using a vernier caliper and found to be ~420 μm.

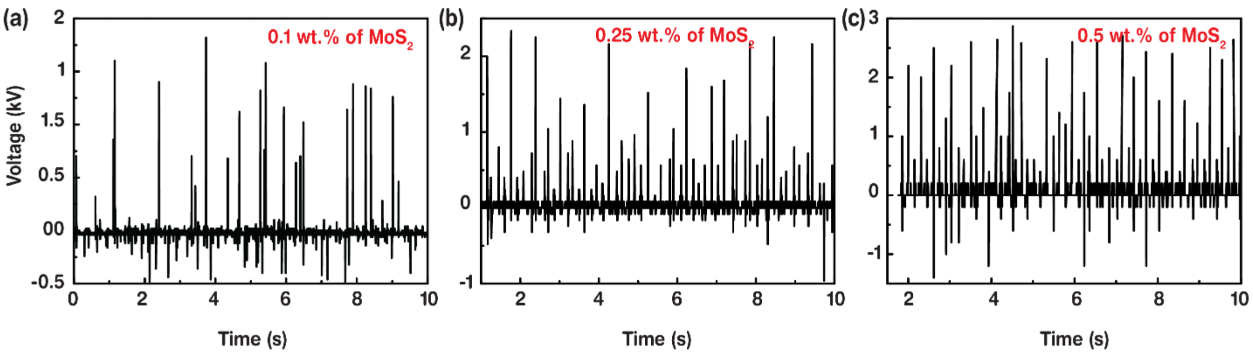

Figure S1. MoS_2 NP-concentration-dependent MoS_2-CQD-CDNA TENG device performance.

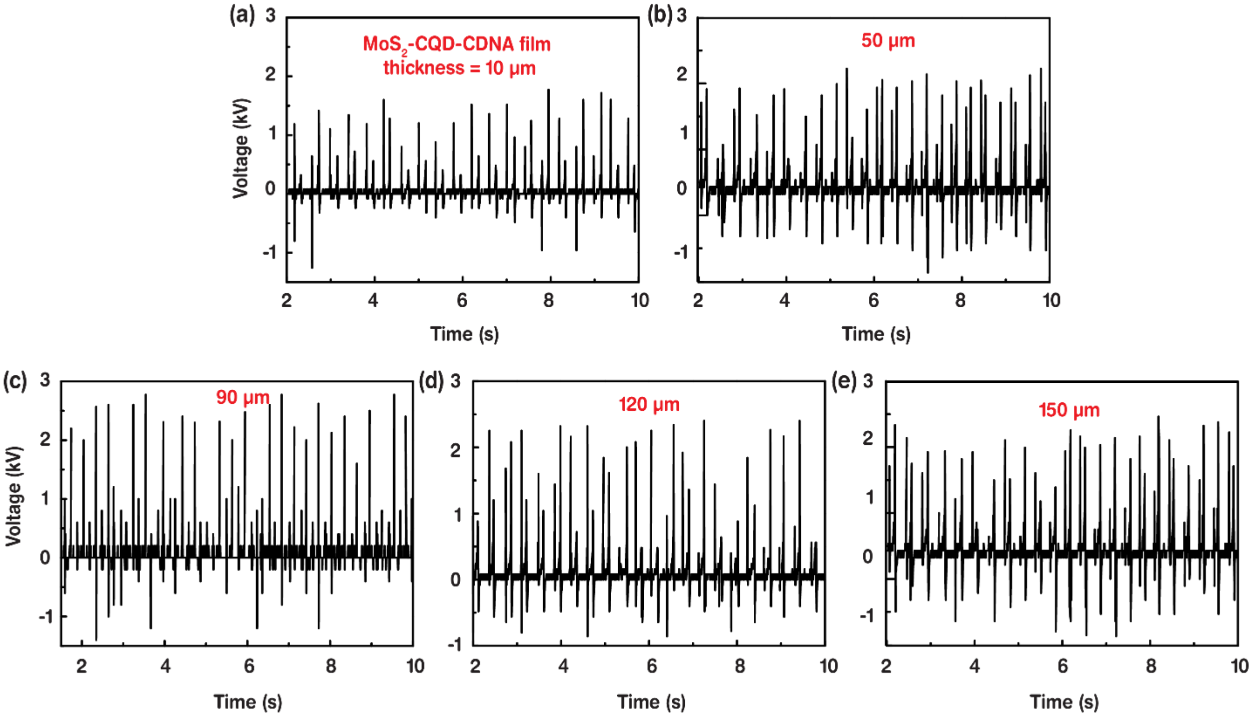

Figure S2. Thickness-dependent performance of the MoS_2-CQD-CDNA thin film-based TENG device.

No.	Band Position (cm^{-1})	Band Assignment
1	527	527 cm^{-1} (sugar phosphate vibrations), 531 cm^{-1} DNA-MoS_2, and 540 cm^{-1} (DNA-MoS_2-CQD)
2	783	Sugar Phosphate Vibration
3	823	Deoxyribose phosphate
4	914	Adenine – Thymine base pairs
5	960	C–C and C–O of deoxyribose skeletal motion
6	1009	P–O or C–O stretching
7	1056	C–O deoxyribose stretching, –C–O–C (MOS_2–CQD)
8	1368	Cytosine and Guanine, –COOH (MOS_2–CQD)
9	1478	Cytosine (in-plane vibrations, –C=C (MOS_2–CQD)
10	1650	Thymine (C2=O stretching), H–O–H (MOS_2–CQD)
11	1700	Guanine (C=O stretching)
12	2800–2975	CH2 and CH3 groups stretching vibrations in the alkyl chains of CTMA
13	3190–3340	OH stretching (DNA and MOS_2–CQD)

Table S1. Fourier transform infrared spectral absorption band positions and corresponding band assignments of the MOS_2-CQD-CDNA thin film.

S2. X-ray photoelectron spectroscopy of the MoS_2-CQD-CDNA thin film

The X-ray photoelectron spectroscopy (XPS) full-survey spectra of the core shell elements such as C, N, O, and P present in the pristine CDNA and MoS_2-CQD-CDNA thin films are shown in **Figure S3**a, **S3**b. The pristine CDNA consists of bases, sugar, and phosphate groups with specific bonds between C, N, O, and P, and the MoS_2-CQD-CDNA thin film consists of Mo and S, obtained by deconvolution of the peaks, in addition to those present in the pristine CDNA. The deconvoluted peaks corresponding to the core shell elements C, N, O, and P present in the pristine CDNA and MoS_2-CQD-CDNA thin films demonstrate chemical shifts, compositional changes, and intensity variations. The C 1s peaks of CDNA are observed at binding energies of 284.83, 286.46, 287.52, and 289.18 eV, which correspond to the functional groups C–C/C=C/C–H, C–O/C–N/N–C=N/N–C–N, N–C–O/N–C=N/N–C=O, and N–C(=O)–N, respectively. Subsequently, the C 1s binding energy peaks of the MoS_2-CQD-CDNA thin films are at 284.79, 286.22, and 287.66 eV, which correspond to C–C/C=C/C–H and

C–N/C=N/C–O/N–C–O/N–C=C, respectively (**Figure 1**k, and **Figure S1**c, **S1**g). The N 1s peaks of the pristine CDNA appear at 398.75, 400.2, and 402.35 eV, corresponding to C–NH_2/C=N–C/N=C, N–C–O/N–C=O, and N–C, respectively, whereas in the case of the MoS_2-CQD-CDNA thin films, the N 1s peaks are visible at 398.83 and 400.13 eV, which can be assigned to N–H–C, C–NH_2/C=N–C/N=C, and N–C, respectively (shown in **Figure 1**l and **Figure S3**d, **S3**h).

Likewise, the deconvoluted O 1s peaks of the CDNA thin films appear at 530.17 and 532.12 eV, which can be ascribed to C–O–C/C=O/P=O and C=O/P=O, respectively. In the case of the MoS_2-CQD-CDNA films, the O 1s peaks appear at 530.85, 532.58, and 535.41 eV, which can be attributed to C–O–C/C=O/P=O, C=O/P=O, and P–O, respectively (**Figure 2**m and **Figure S1**e, **S1**i). Finally, the P 2p characteristic peak of the CDNA thin film can be deconvoluted into two peaks at 132.85 and 133.65 eV, which are associated with the chemical compositions P=O/P–O^- and P $2p_{3/2}$/PO_4^-/P–O, respectively. By contrast, the deconvoluted P 2p peaks of the MoS_2-CQD-CDNA thin film appear at 132.97 and 133.53 eV, which indicate the chemical assignments P=O/P–O^- and P$2p_{3/2}$/PO_4^-/P–O, respectively (**Figure 1**n and **Figure S1**f, **S1**j). The individual core-shell elemental peak intensities either increase or decrease due to the variations in the atomic percentages of the respective elements, which occur when the MoS_2, CQD, and CDNA molecules interact with each other. The physical parameters such as peak height, full width at half maximum (FWHM), area, and atomic percentage with respect to the binding energies changes in the pristine CDNA and MoS_2-CQD-CDNA films were obtained from the respective deconvoluted XPS spectra as shown in **Table S2**.

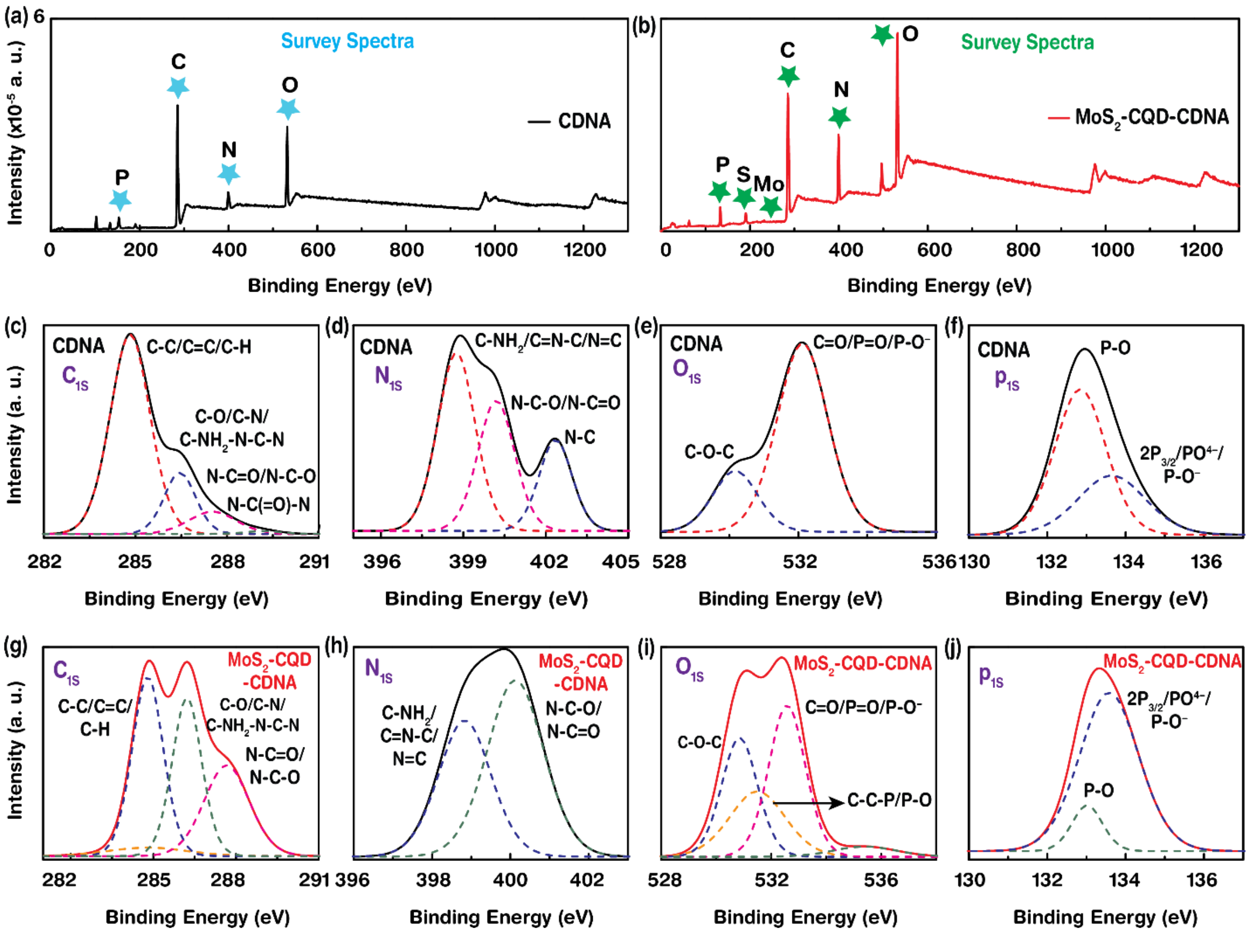

Figure S3. XPS survey spectra, deconvolved individual elemental analysis of the pristine CDNA and MoS_2-CQD-CDNA thin films. (a, b) XPS full-survey spectra represents the core elements present in the pristine CDNA and MoS_2-CQD-CDNA thin films. (c-f) and (g-j) Gaussian fitting curves displayed with different peak positions present in the representative regions (C 1s, N 1s, O 1s, and P 2p) in the spectra of the CDNA and MoS_2-CQD-CDNA thin films, respectively. The binding energies and the corresponding functional groups are denoted for each deconvolved spectral element.

CDNA / MoS_2-CQD-CDNA	Peak (B.E)		Height (CPS)		FWHM (eV)		Area (P) (CPS • eV)		Atomic %	
**Mo3d**		232.04	-	635.76	-	0.85	-	2835.75	-	1.06
**S2p**		163.36	-	343.97	-	0.84	-	1619.8	-	0.21
**C1s**	284.83	232.04	53531.97	54025.52	1.66	3.67	123393	200260.4	69.98	53.89
**N1s**	399.11	399.65	6882.14	34657.62	2.81	2.62	24881.8	93681.52	9.09	15.75
**O1s**	532.1	531.93	32511.5	67882.7	1.79	3.19	78793.7	235332.8	18.49	25.41
**P2p**	132.96	133.34	3043.86	10742.92	1.86	1.79	6382.9	20977.97	2.44	3.68

Table S2. Quantitative analysis of the various physical parameters based on the survey spectra of the pristine CDNA and MoS_2-CQD-CDNA thin films. The numerical values of the binding energies with the corresponding peak positions, peak heights, FWHM, peak areas, and peak atomic % are shown in **Table S2**.

S3. Charge transfer mechanism between CDNA, MoS_2, and CQDs

The detailed charge transfer mechanism of the MoS_2-CQD-CDNA film under ultraviolet (UV)–visible and near-infrared light illuminations can be understood by the energy band diagram (**Figure 4**d). The electron affinity and energy bandgap of CQDs (χ = ~3.52 and E_g = 3.33 eV) and MoS_2 NPs (χ = 4 and E_g = 1.53 - 4 eV) respectively, measured elsewhere. The discrete distribution of CQDs with MoS_2 NPs in CDNA molecules are reaching its equilibrium conditions, where electrons flow from CQDs towards MoS_2 which results in alignment of Fermi level. Under UV light, the absorption of photons by the localized π electrons result in electron–hole pairs, and due to the radiative recombination of these photogenerated electrons and holes, a blue emission is observed. Hence, in case of the pristine CQD, the photogenerated electron–hole

pairs radiatively recombine, which hinders the performance of the photodetector. Thus, MoS_2 is required as a transport material, whose energy levels are well adjusted such that the radiative recombination can be prevented, and the photogenerated electron–hole pairs can be easily captured. In this case, due to the presence of MoS_2, whose conduction band is lower than the lowest unoccupied molecular orbital (LUMO) of CQD, and the valence band is above the highest occupied molecular orbital (HOMO) level, the photogenerated electron–hole pair transports to MoS_2 before reaching the HOMO level of CQD. Since both the contacts are on MoS_2, the electron–hole pairs are separated because of the electric field generated at the unipolar junction created between MoS_2 and CQD. Even though CQD was found to be electron donor by zetapotential measurements conducted elsewhere, the Fermi level difference between MoS_2 and CQD favors the formation of unipolar junctions, whose barrier potential is the difference between the Fermi levels of MoS_2 and CQD (**Figure 4**d). Additionally, there were numerous such unipolar junctions which cumulatively add up and aid in creating an effective electric field, thereby assisting in electron–hole separation; they also increase the photocurrent. Notably, if one of the contacts is taken from CQD, then the photogenerated holes are trapped in MoS_2, which might cause recombination of the photogenerated electrons. Under visible light illumination, electron–hole pairs are generated in MoS_2, where the presence of extra electrons causes a change in the Fermi level, which further increases the barrier height of MoS_2–CQD. The increase in the barrier height helps in preventing the recombination of the photogenerated electron–hole pairs. Since the conduction band of MoS_2 is lower than the LUMO of CQD and the valence band of MoS_2 is higher than the HUMO of CQD, the photogenerated electrons remain in MoS_2 and are transported to the metal contacts due to the localized electric field generated by the barrier potential created between MoS_2 and CQD. Even though CQD was discretely distributed with

MoS_2, a larger portion of MoS_2 was exposed to illumination, which led to higher responsivity values under visible illumination. Therefore, the role of CQD is twofold, i.e., it absorbs UV photons and generates a local electric field, which helps in an efficient charge separation under visible and near-infrared illumination. This mechanism is vital for enhancing the MoS_2-CQD-CDNA-based TENG device *V*oc responses. These results indicate that the prepared MoS_2-CQD-CDNA-based TENG device can be operated under normal conditions under visible light stimulations.

Ref.	Structure & Materials	*I*sc (*J*sc)	*Vo* (V)	Power density
1	ITO/PDMS/P(VDF-TrFE)/AgNPs/ MoS2/PPy+Pt NPs/PET	120 μA (120 μA/cm^2)	200	14.4 mW/cm^2
2	Cu/Nylon/MoS2/Cu	0.82μA (1.04 μA/cm^2)	7.5	7.8 μW/cm^2
3	Al/PI-MoS2 composites /Al	18 μA/cm2	400	2.57 mW/cm^2
4	Al/Graphite/paper/MoS2 /Al	0.15 μA (0.025 μA/cm^2)	10	0.255 μW/cm^2
**5**	**CNT/MoS_2-CQD-CDNA/PDMS (This work)**	***I*sc: Av. ~3.25 μA (Max. ~3.5 μA) *J*sc: Avg. ~2.16 μA/cm^2 (Max. ~2.33 μA/cm^2**	**Avg. 1.6 kV (Max. ~2.5 kV)**	**275 mW/cm^2**

Table S3. Comparison of the electrical output performance of previously fabricated contact separation mode driven TENGs with that prepared in our study.

1. M. Kim, C. Lee, S. H. Kim, M. U. Park, J. Yang, Y. Yi, K. H. Yoo, *J. Mater. Chem. A*. **2021**, *9*, 10316-10325.
2. M. Seol, S. Kim, Y. Cho, K. E. Byun, H. Kim, J. Kim, S. Park, *Adv. Mater.* **2018**, *30*, 1870294.
3. C. Wu, T. W. Kim, J. H. Park, H. An, J. Shao, X. Chen, Z. L. Wang, *ACS nano,* **2017**, *11*, 8356-8363.
4. S. Karmakar, R. Sarkar, C. S. Tiwary, P. Kumbhakar, *J. Alloy. Compd.* **2020**, *844*, 155690.

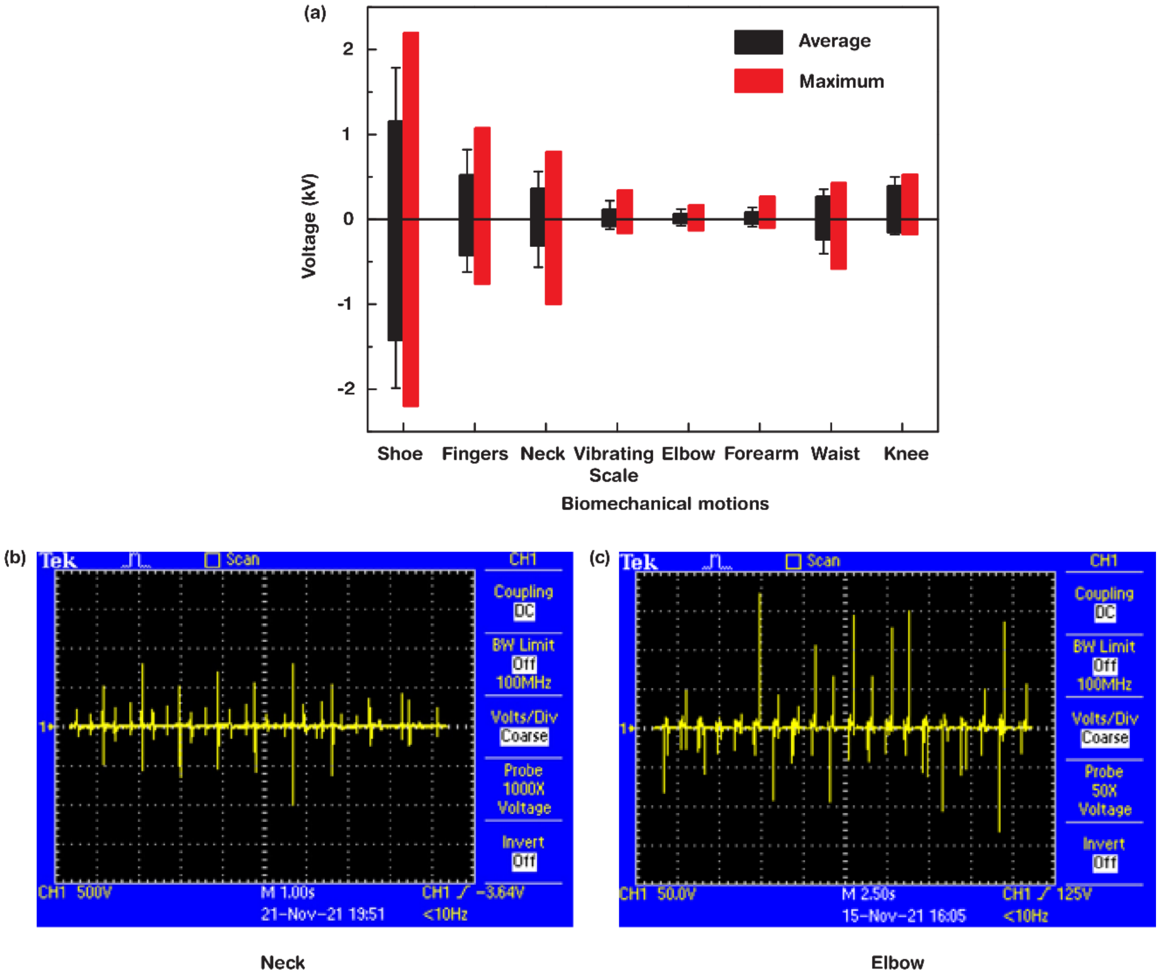

Figure S4. MOS_2-CQD-CDNA TENG device performance under human biomechanical motions. a) The average and maximum voltages generated under biomechanical motions such as shoe, finger, neck, vibration, elbow, forearm, waist, and knee motions. b,c) Oscilloscope screens showing the voltage response with time for neck and elbow biomechanical motions.

**Light illumination wavelength (nm)**	**Power density (mW/cm^2)**	**Rise time (τ_r)**	**Fall time (τ_f)**
**365**	**6.5**	**39.30**	**36.10**
**405**		**45.13**	**39.88**
**617**		**36.89**	**30.15**
**730**		**40.21**	**36.23**

Table S4: Quantitative analysis of the calculated rise time (τ_r) and fall time (τ_f) measured by photostimulation of the MOS_2-CQD-CDNA film under different light wavelengths such as 365, 405, 617, and 730 nm.

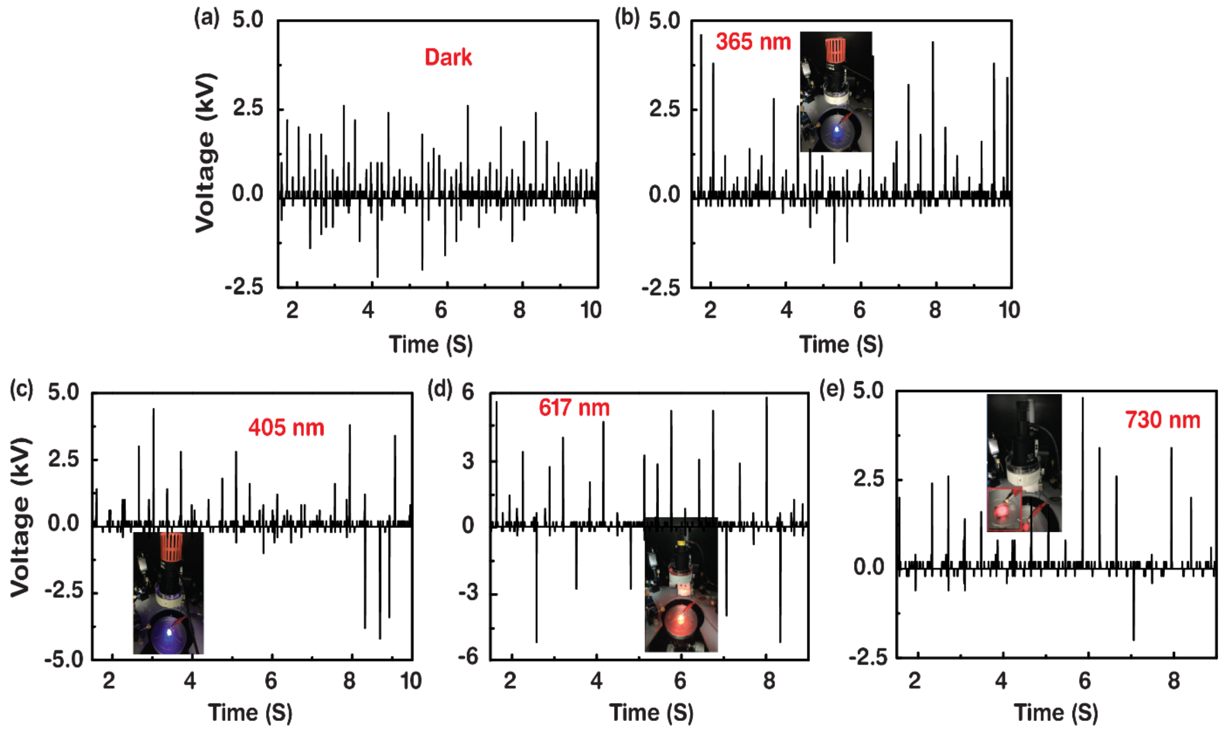

Figure S5. Open-circuit voltage of the MoS_2-CQD-CDNA TENG device and its enhanced response with photostimulation under different LED wavelengths (*i.e.*, 365, 405, 617, and 730 nm). Each insert shows the measurement set up with light illumination on the MoS_2-CQD-CDNA TEGN device.

S. No.	Photostimulation (nm)	Average *V*oc (kV)	Maximum *V*oc (kV)
1	Dark	1.2	2.6
2	365	1.6	4.7
3	405	1.5	4.4
4	617	2.4	5.8
5	730	2.1	4.8

Table S5: Photostimulation response of the MoS_2-CQD-CDNA TENG device under varied light wavelengths. Here, we display both the average and maximum *V*oc responses.

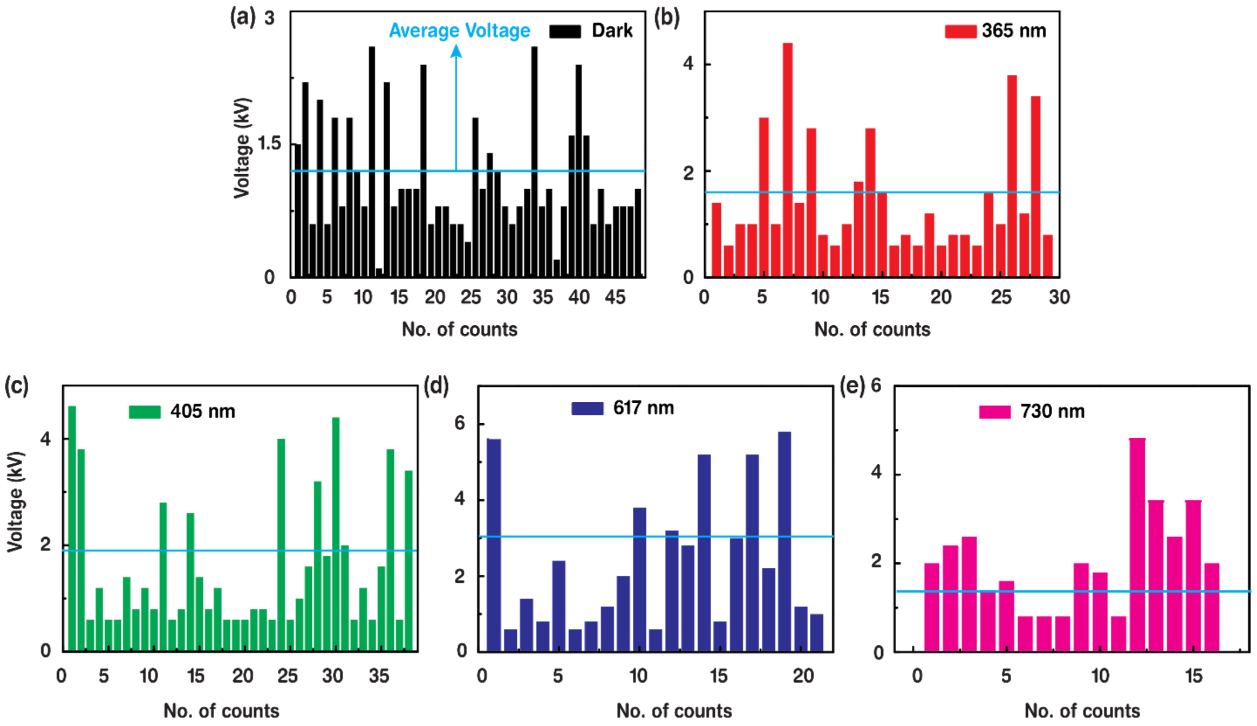

Figure S6. Statistical analysis of the MoS_2-CQD-CDNA TENG device operating in (a) dark, (b) 365, (c) 405, (d) 617, and (e) 730 nm light illuminations. Blue lines indicated average voltages shown in Figure 4e.

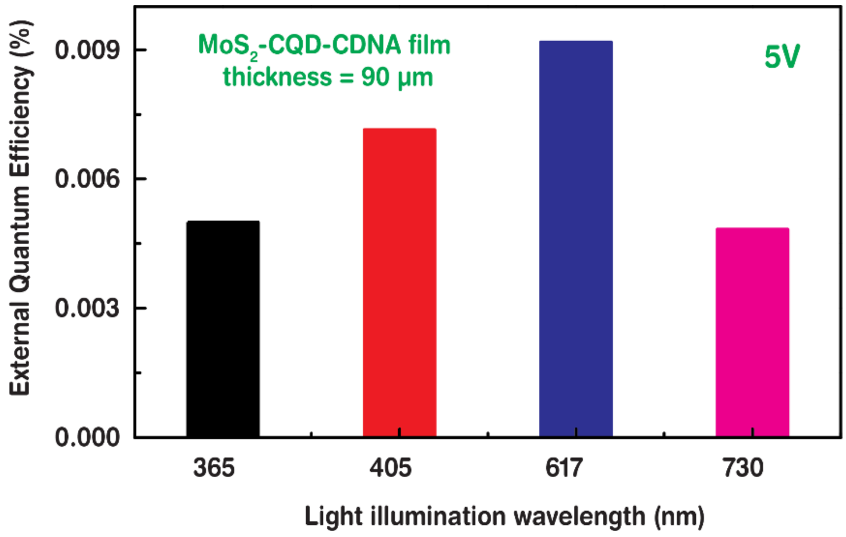

Figure S7. External quantum efficiencies (EQEs) of the MoS_2-CQD-CDNA nanocomposite device under the 365, 405, 617, and 730 nm light illuminations.

Figure 4b displayed the responsivity (R), which was calculated by $R = I_{ac.ph} / PA$ where $I_{ac.ph} = I_{ph.max} – I_{d.max}$ was actual photocurrent obtained by removing the dark current ($I_{d.max}$) from the photocurrent ($I_{ph.max}$), P referred to power density of light, and A was the contact area of the sample. As shown in Figure S7, we obtained the external quantum efficiency (EQE (%) = $(Rhc/q\lambda) \times 100$, where R, h, c, q, λ referred the responsivity, plank constant, speed of light, electron charge and wavelength, respectively). The photon flux was directly proportional to the light intensity. EQE values were determined from the R-values depending on the light wavelength (λ). We obtained EQE results of 0.00499% for 365 nm, 0.00715% for 405 nm, 0.00918 % for 617 nm, and 0.0049 % for 730 nm at the fixed power density of 6.5 mW/cm^2. The obtained Rs and EQEs showing the trend as 730 < 365 < 405 < 617 nm indicated that our MoS_2-CQD-CDNA TENG device was more responsive to visible light, compared to UV and NIR illumination. This work helps not only for energy harvesting device but also for flexible broadband photodetector applications in the field of flexible and wearable electronics and optoelectronics.

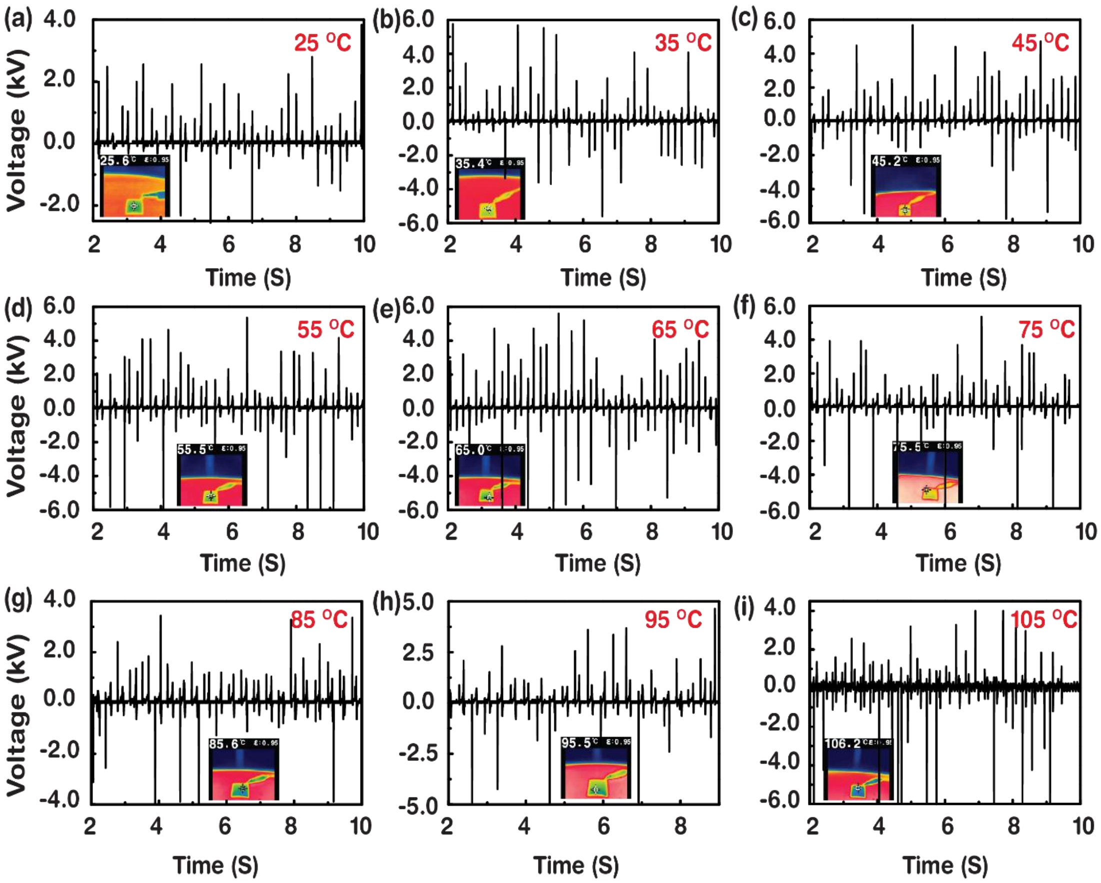

Figure S8. MoS_2-CQD-CDNA TENG device performance at different temperatures in the range of 25–105 °C with 10 °C interval. Each graph shows the output voltage dependence at different temperatures such as a). 25 °C, b). 35 °C, c). 45 °C, d). 55 °C, e). 65 °C, f). 75 °C, g). 85 °C, h). 95 °C, and i). 105 °C. Each insert shows the temperature measured by an FLIR, ETS320 thermal imaging camera at the time of triboelectrification.

**No.**	**Temperature (°C)**	**Average Voc (kV)**		**Maximum *Voc* (kV)**	
**1**	25	1.26	-1.0	3.84	-3.44
**2**	35	1.71	-2.0	5.76	-5.6
**3**	45	2.07	-1.67	5.68	-5.76
**4**	55	1.95	-1.18	5.36	-3.12
**5**	65	2.33	-1.75	5.6	-5.68
**6**	75	1.87	-2.05	5.36	-3.44
**7**	85	1.32	-0.84	344	-3.12
**8**	95	1.45	-1.0	4.64	-4.24
**9**	105	1.50	-1.37	4.0	-5.6

Table S6: Temperate modulation response of the MoS_2-CQD-CDNA TENG device at different temperatures.

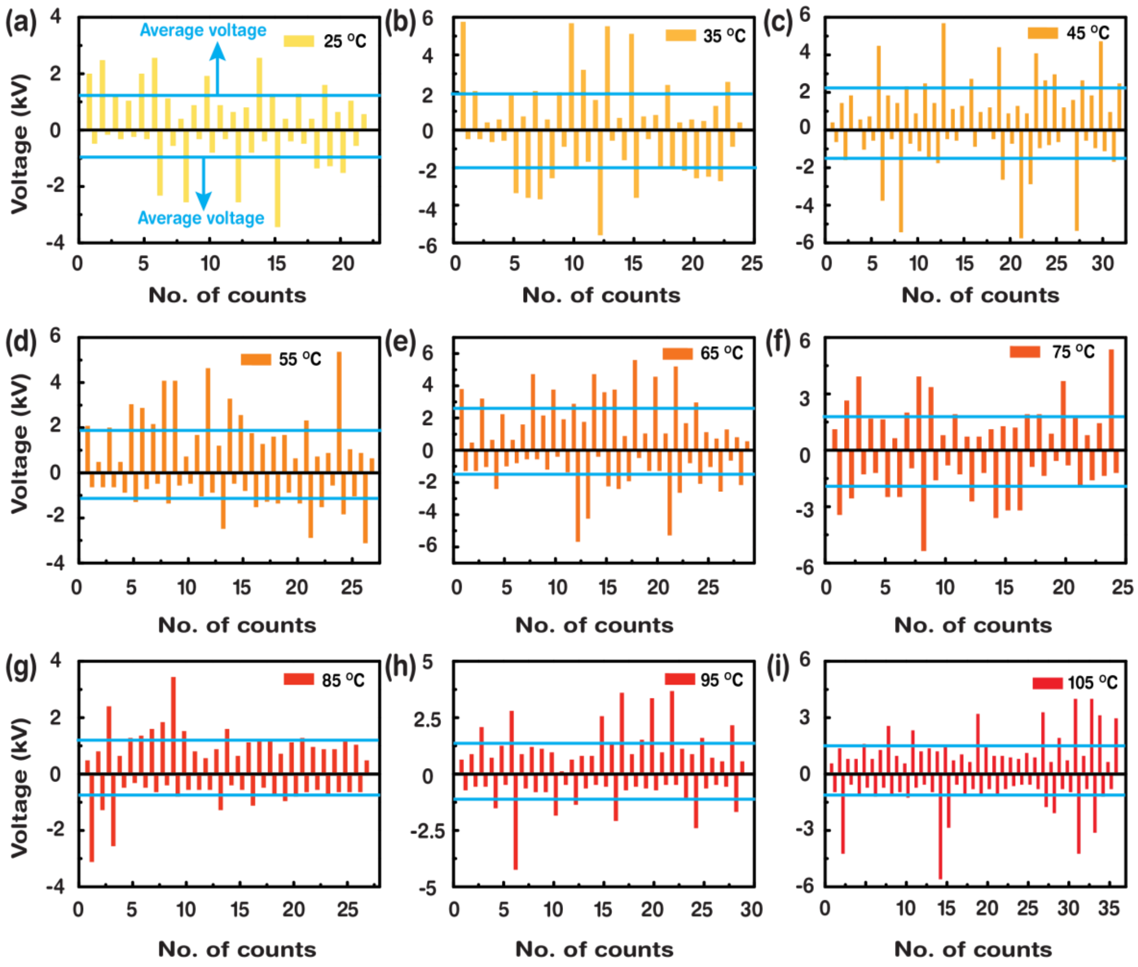

Figure S9. Statistical analysis of the MoS_2-CQD-CDNA TENG device at different temperatures in the range of 25–105 °C with the 10 °C interval. Each plot shows the output voltage value measured at different temperatures of (a) 25 °C, (b) 35 °C, (c) 45 °C, (d) 55 °C, (e) 65 °C, (f) 75 °C, (g) 85 °C, (h) 95 °C, and (i) 105 °C. Temperature values were measured using a thermal imaging camera during the triboelectric experiments. Blue lines indicated average voltages shown in Figure 4f.

Movies

Movie S1. Activation of LEDs

Movie S2. Biomechanical motions
